# New species of *Rogmocrypta* Simon, 1900 from New Caledonia, with remarks on relationships and distribution (Araneae, Salticidae)

**DOI:** 10.3897/zookeys.697.13381

**Published:** 2017-09-14

**Authors:** Barbara M. Patoleta, Joanna Gardzińska, Marek Żabka

**Affiliations:** 1 Siedlce University of Natural Sciences and Humanities, Faculty of Natural Science, Department of Zoology, Prusa 12, 08-110 Siedlce, Poland

**Keywords:** distribution modelling, jumping spiders, Pacific Islands

## Abstract

Five new species of *Rogmocrypta*: *R. karolinae* (♀), *R. koniambo* (♀), *R. patryki* (♀), *R. raveni* (♀), and *R. rollardae* (♀) are diagnosed, described, and illustrated. The definition of the genus is ammended and its distribution and relationships are discussed.

## Introduction

The fauna of New Caledonia is often discussed in terms of Gondwanan heritage. Indeed, the island group was separated from Gondwana some 85 MYA, but later experienced multiple subductions and submergences (Cluzel et al. 2012) and, in fact it only emerged in post-Eocene (37 MYA). Consequently, New Caledonian biota, fauna, and flora should not be discussed in terms of direct *Gondwanan heritage*, but rather as the result of local radiations and colonisation from other sources (Keast and Miller 1996, Grandcolas et al. 2008, Heads 2008, 2010, 2014). The phenomenon of local radiation is also known for several salticid genera such as *Corambis* Simon, 1901, *Penionomus* Simon, 1903 and *Rhondes* Simon, 1901 (Maddison et al. 2008); all are part of the Australasian Astioida clade and derived from Australian ancestors between 9 and 20 MYA (Bodner and Maddison 2012). The genus *Rogmocrypta* (here) with seven nominal species is also the case of radiation *in situ*.

Our initial aim is to present a complete revision of the genus; however, the lack of type material for *R. nigella* Simon, 1900 and *R. puta* Simon, 1900 limited our goals.

## Materials and methods

The material was obtained from the following collections:

**MNHN** Museum National d’Histoire Naturelle, Paris, France

**QM** Queensland Museum, Brisbane, Australia.

The examination specimen methods were as described by Żabka (1991). The drawings were made using a grid system. The photographs were taken with Nikon D5200 camera and Nikon SMZ1000 stereomicroscope, and were digitally processed with ZoomBrowser and HeliconFocus software. The dissected epigynes were digested in 10% KOH and studied under compound microscope. The actual and predicted distributional maps were generated with DIVA-GIS bio-climatic software using BIOCLIM application (Nix, 1986; Busby, 1991). Our model has been produced with 14 field records and met the requirements for the software (at least 5–10 records; Hernandez et. al. 2006). The following environmental variables were used in the analysis: annual mean temperature, mean monthly temperature range, isothermality, temperature seasonality, max temperature of warmest month, min temperature of coldest month, temperature annual range, mean temperature of wettest quarter, mean temperature of driest quarter, mean temperature of warmest quarter, mean temperature of coldest quarter, annual precipitation, precipitation of wettest month, precipitation of driest month, precipitation seasonality, precipitation of wettest quarter, precipitation of driest quarter, precipitation of warmest quarter, precipitation of coldest quarter.

**Abbreviations used in the text and figure legends are**:

**AEW** anterior eye width,

**AME** anterior medial eyes,

**AL** abdomen length,

**AW** abdomen width,

**cd** copulatory duct,

**CH** cephalothorax height,

**CL** cephalothorax length,

**co** copulatory opening,

**CW** cephalothorax width,

**EFL** eye field length,

**e** embolus,

**eo** endites outgrowth,

**fd** fertilisation duct,

**L** leg,

**PEW** posterior eye width,

**PLE** posterior lateral eyes,

**PME** posterior medial eyes,

**rta** retrolateral tibial apophysis,

**s** spermatheca,

**t** tegulum,

**tr** transverse ridge.

## Taxonomy

### Genus Rogmocrypta

Taxon classificationAnimaliaAraneaeSalticidae

Simon, 1900

Rogmocrypta Simon, 1900: 387; 1901: 389, 445–446; Maddison et al. 2008: 52–55; Maddison 2015: 277.

#### Type species.

*R. elegans* (Simon 1885) = *Chalcoscirtus elegans* Simon 1885, originally designated by Simon (1900).

#### Diagnosis.

**Figures 1–9.**
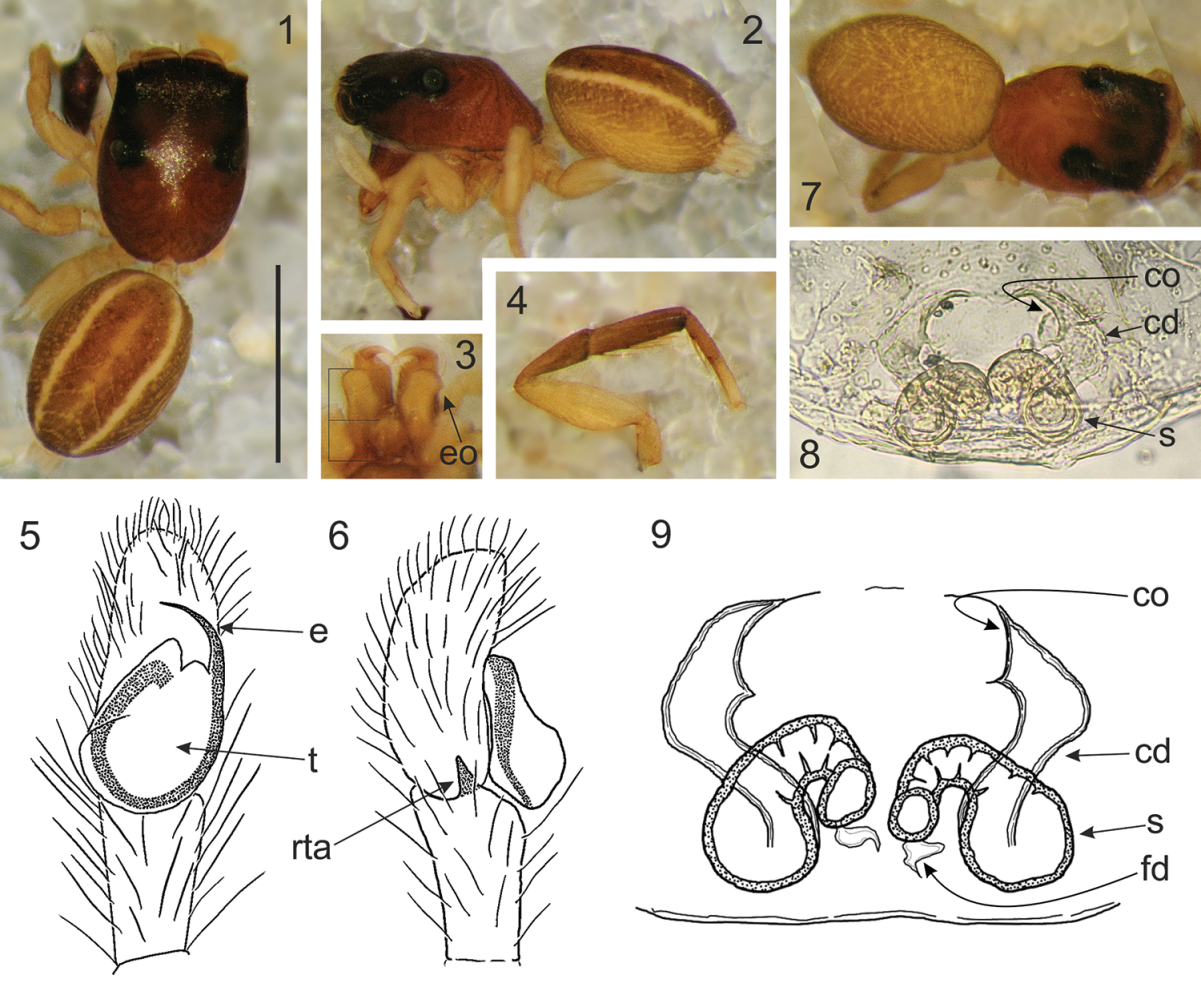
*Rogmocrypta elegans*. **1–6** Male (holotype) **1** Dorsal view **2** Lateral view **3** Endites and labium **4** First leg **5** Right palp ventrally **6** Same, retrolaterally **7–9** Female (paratype) **7** Dorsal view **8–9** Vulva. Abbreviations: cd: copulatory duct, co: copulatory opening, e: embolus, eo: endites outgrowth, fd: fertilisation duct, rta: retrolateral tibial apophysis, s: spermatheca, t: tegulum. Scale bar: 1 mm.

**Figures 10–15.**
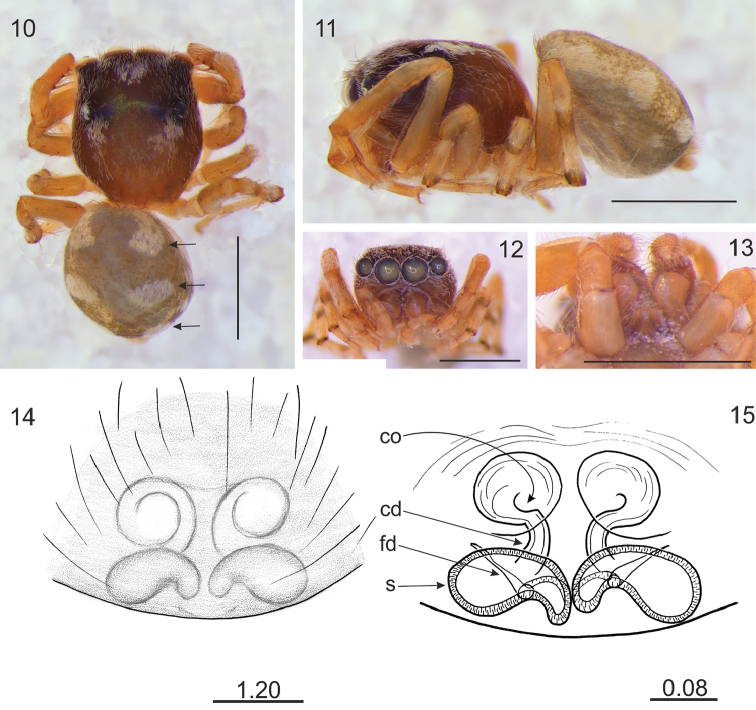
*Rogmocrypta karolinae* sp. n. (female holotype). **10** Dorsal view (arrows indicate patches of white scales being distinctive diagnostic characters) **11** Lateral view **12** Frontal view **13** Endites and labium **14** Epigyna **15** Vulva. Abbreviations: cd: copulatory duct, co: copulatory opening, fd: fertilisation duct, s: spermatheca. Scale bars: 1 mm (**10–13**); 1.20 mm (**14**); 0.08 mm(**15**).

**Figures 16–21.**
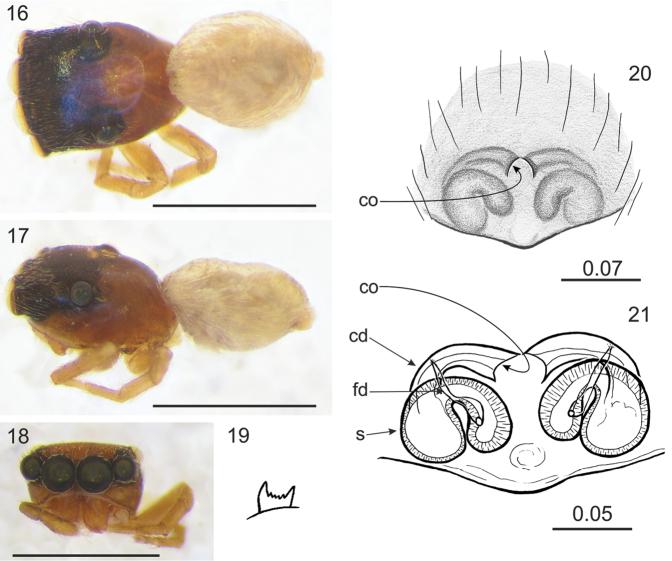
*Rogmocrypta koniambo* sp. n. (female holotype). **16** Dorsal view **17** Lateral view **18** Frontal view **19** Retromarginal tooth **20** Epigyna **21** Vulva. Abbreviations: cd: copulatory duct, co: copulatory opening, fd: fertilisation duct, s: spermatheca. Scale bars: 1 mm (**16–18**); 0.07 mm (**20**); 0.05 mm (**21**).

Differs from related genera by tiny or small body size. Unlike in *Lystrocteissa* (Patoleta and Gardzińska 2013, figs 9–15) the habitus is not ant-mimic (Figs 1, 7, 10, 16, 28) and much more compact than in *Corambis* (Szűts 2002, figs 1, 10–12). Male palpal embolus is sabre-like (Fig. 5) and shorter than in *Penionomus* (Żabka 1988, fig. 114) and in some species of *Rhondes* (Patoleta 2016, figs 9–14). Tegulum without lobe (more or less marked in relatives). Seminal duct not meandering, tibial apophysis short (Fig. 6). Unlike in *Rhondes* (Patoleta 2016, figs 22–27). Epigyne with no central pocket (Figs 8, 14, 20, 25, 34). Copulatory ducts much shorter than in *Penionomus* (Żabka 1988, fig. 118) and not twisted (Figs 9, 15, 21, 27, 36, 43). Accessory glands not distinctive - unlike in *Corambis* (Szűts 2002, figs 4, 17) where they are long.

#### Description.

Cephalothorax medium-high, longer than broad and widest at the level of coxae II; fovea in distinct depression, posterior slope steep, starting behind fovea, eye field wider than long, trapezoid (PLE<ALE). Eyes in three rows, the first row straight. Chelicerae with two promarginal teeth, retromarginal tooth 4–6-cuspidate (Figs 19, 33, 41). Endites slender and divergent, in male with lateral outgrowth (Fig. 3). Labium wider than long. Sternum longer than wide. Abdomen ovoid, longer than wide. Spinnerets short. Legs moderately long and thin. Leg formula: I–IV–II–III. Male palpal organ simple: cymbium unmodified, tegulum longer than wide, ovoid, with no lobes, embolus curved, rather thin, retrolateral tibial apophysis single (Fig. 6). Epigyne copulatory openings located close to each other (Figs 21, 27, 43) or distinctly separated (Figs 9, 15, 36), sometimes strongly sclerotised (Figs 25–27). Copulatory ducts narrow. Spermathecae C-shaped (Figs 9, 15, 36) or semicircular (Figs 21, 27, 43).

#### Distribution.

According to WSC (2017) three species of *Rogmocrypta* are listed from New Caledonia (*R. elegans*), Philippines (*R. nigella* Simon, 1900) and Singapore (*R. puta* Simon, 1900). However, two latter are poorly known, their bioclimatic distributional predictions (Fig. 45) do not match *Rogmocrypta*-pattern and they probably are not congeneric. Additionally, the five species described here seem to confirm New Caledonia as the diversity and radiation centre.

#### Biology.

The species treated here are litter dweller in humid forests.

#### Remarks.

According to recent molecular studies (Maddison et al. 2008, Maddison 2015), *Rogmocrypta* belongs to Viciriini tribe within the Australasian Astioida clade and is closely related to other New Caledonian genera such as *Trite* Simon, 1885, *Penionomus* Simon, 1903 and *Lystrocteissa* Simon, 1884. However, the analysis of male genitalia here and in Maddison et al (2008: fig. 3) raises some doubts about congeneric status of *R. elegans* (we dealt with the type) and cf. *Rogmocrypta* sp. in Maddison et al. (2008): both show important differences in embolus structure and tegular lobe, which is missing in *R. elegans*. To clarify the relationships of *Rogmocrypta* it is necessary to perform molecular tests for all species ever listed in the genus. At this stage any reference to other New Caledonian genera as possible relatives can only be provisional.

### Rogmocrypta elegans

Taxon classificationAnimaliaAraneaeSalticidae

(Simon, 1885)

Figures 1–9 , 44

Chalcoscirtus elegans Simon, 1885: 90.
Rogmocrypta elegans : Simon 1901: 445–446, figs 506D–E; Prószyński 1984: 123–124.

#### Material.

1♂ holotype, 1♀ paratype, New Caledonia: Nouméa, MNHN Paris, nr 7527.

#### Diagnosis.

Males abdomen with two whitish stripes (Figs 1–2), embolus curved, arising from antero-prolateral part of tegulum (Fig. 5), retrolateral tibial apophysis short and conical (Fig. 6). Epigyne copulatory openings oriented towards each other, more separated than in *R. koniambo* sp. n.

#### Distribution.

Known only from the type locality (Fig. 44).

#### Remark.

This is the only known and illustrated species of *Rogmocrypta* (Prószyński 1984), and it is used here for comparative purposes.

### Rogmocrypta karolinae sp. n.

Taxon classificationAnimaliaAraneaeSalticidae

http://zoobank.org/184FCC48-B4F4-4034-B68B-68B2F3BF1D8A

Figures 10–15 , 44

#### Material.

1♀ holotype, New Caledonia: Mandjélia (164°32'E, 20°24'S), 600m elev., rainforest, pitfalls, October 1992–17 February 1993, Raven R, Guillbert E, QM S44894; 1♀, paratype, same data as holotype; 3♀, New Caledonia: Mandjélia (164°32'E 20°24'S), pitfalls, 13 May–October 1992, Raven R, Guillbert E, Ingram G, QM S37722.

#### Etymology.

For Karolina, daughter of Joanna Gardzińska.

#### Diagnosis.

Cephalothorax and abdomen with distinctive patches of white scales (Figs 10–11). Copulatory openings closer to each other than in *R. elegans* (Fig. 15). Spermathecae horizontal (Figs 14–15).

#### Description.

Female holotype. Cephalothorax brown with darker cephalic part, with patches of scales (Fig. 10). Abdomen greyish brown, with three pairs of patches covered with white scales. Spinnerets whitish. Chelicerae with single retromargin 5-cuspidate tooth. Clypeus narrow (17% of AME diameter), covered with sparse white hairs. Labium and endites brown with lighter chewing margins. Sternum and venter greyish brown. Legs light brown, tibiae and metatarsi with darker bands (Fig. 12), metatarsi and patellae covered by white scales. Epigyne copulatory openings oriented towards each other, copulatory ducts sinuous, spermathecae C-shaped, close to each other (Fig. 15). Dimensions. CL 1.23, CW 0.95, CH 0.54, EFL 0.55, AEW 0.97, PEW 0.85, AL 1.15, AW 1.05, LI: 3.42, LII: 1.99, LIII: 1.70, LIV: 2.61.

Male unknown.

#### Distribution.

Known from type locality only (Fig. 44).

### Rogmocrypta koniambo sp. n.

Taxon classificationAnimaliaAraneaeSalticidae

http://zoobank.org/FF2E8207-FCA3-4854-8F15-BABDE1AE13AF

Figures 16–21 , 44

#### Material.

1♀ holotype, New Caledonia: Mt. Koniambo (164°47'11"E, 20°59'42"S), 700m elev., forêt seche/rub, A&S Tillier, 25 March 1987, MNHN.

#### Etymology.

The name refers to the type locality.

#### Diagnosis.

In comparison to previous species copulatory openings closer to each other and located just in front of spermathecae.

#### Description.

Female holotype (in bad condition). Cephalothorax brown with darker cephalic part, covered with sparse white scales. Foveal depression well marked (Fig. 16). Abdomen ovoid, pale, covered with sparse white scales. Spinnerets whitish. Palps and legs II and III greyish brown. Other legs missing. Chelicerae short, brown, retromarginal tooth 6-cuspidate (Fig. 19). Labium brown, endites light brown with whitish chewing margins. Venter whitish. Epigyne copulatory ducts and spermathecae semicircular, the latter almost horizontal (Fig. 21). Dimensions: CL 1.06, CW 0.77, CH 0.42, EFL 0.51, AEW 0.80, PEW 0.65, AL 0.93, AW 0.74, LI and LII missing, LIII: 1.58, LIV: 1.72.

Male unknown.

#### Distribution.

Known from type locality only (Fig. 44).

### Rogmocrypta patryki sp. n.

Taxon classificationAnimaliaAraneaeSalticidae

http://zoobank.org/550946C1-6C62-417A-815C-68E1E0D0C870

Figures 22–27 , 44

#### Material.

1♀ holotype, New Caledonia: Mt. Oua Tilou (164°51'28"E, 20°51'57"S), arête S, 510 m elev., foret sèche, berlesate, st. 198a, 19 October 1988, A&S Tillier, Chazeau J, MNHN; 1♀ paratype same data as holotype; 1♀, New Caledonia: Mt. Panié, 450–950m, 14 May 1984, Monteith G, Cook D, QM S35668; 1♀, New Caledonia: Mt. Panié summit, 1628 m elev., 15 May 1984, Monteith G, Cook D, QM S35665; 2♀, 1 juv. New Caledonia: Mt. Oua Tilou (164°51'28"E, 20°51'57"S), arête S, 510m elev., foret sèche, berlese, st. 198a, 19 October 1988, A&S Tillier, Chazeau J, MNHN.

**Figures 22–27.**
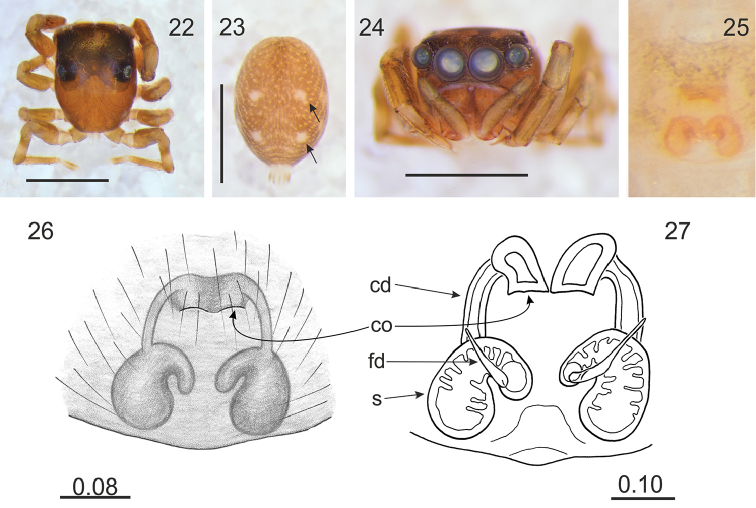
*Rogmocrypta patryki* sp. n. (female holotype). **22** Cephalothorax, dorsal view **23** Abdomen, dorsal view (arrows indicate white patches being distinctive diagnostic characters) **24** Frontal view **25–26** Epigyna **27** Vulva. Abbreviations: cd: copulatory duct, co: copulatory opening, fd: fertilisation duct, s: spermatheca. Scale bars: 1 mm (**22–24**); 0.08 mm (**26**); 0.10 mm (**27**).

#### Etymology.

For Patryk Patoleta, Barbara Patoleta’s son.

#### Diagnosis.

Abdomen with lighter chevrons and distinctive white patches (Fig. 23). Copulatory openings close to each other, orientated posteriorly, and located well anteriorly to spermathecae (Figs 25–27).

#### Description.

Female holotype. Cephalothorax brown, with darker eye field and foveal depression (Fig. 22). Abdomen greyish brown, with distinctive pattern as in Fig. 23. Spinnerets light brown. Cheliceral retromarginal tooth 5-cuspidate. Clypeus brown, much narrower (6%) than AME diameter. Labium and endites light brown. Sternum brown. Venter whitish, with brownish spots. Legs brown, tibiae and metatarsi with darker bands (Figs 22, 24). Epigyne copulatory ducts and spermathecae semicircular, the latter in diagonal position (Figs 26–27). Dimensions. CL 1.40, CW 1.05, CH 0.65, EFL 0.63, AEW 1.00, PEW 0.95, AL 1.53, AW 1.08, LI: 3.06, LII: 2.42, LIII: 2.56, LIV: 3.37.

Male unknown.

#### Distribution.

Known from Mt. Panié and Mt. Oua Tilou in New Caledonia (Fig. 44).

### Rogmocrypta raveni sp. n.

Taxon classificationAnimaliaAraneaeSalticidae

http://zoobank.org/8A020BFF-2D8F-4D8F-9EDA-510F8FB8E22C

Figures 28–36 , 44

#### Material.

1♀ holotype, New Caledonia: Mt. Panié (20°35'S, 164°45'E), 400m elev., pitfalls, October 1992 – February 1993, Raven R, Guillbert E, Ingram G, QM S35759; 2♀♀ paratypes, same data as holotype.

**Figures 28–36.**
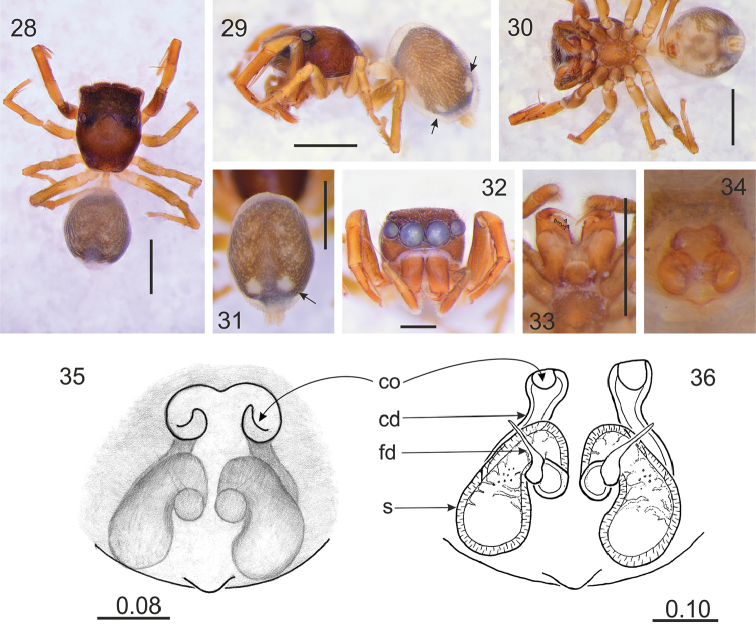
*Rogmocrypta raveni* sp. n. (female holotype). **28** Dorsal view **29** Lateral view **30** Ventral view **31** Abdomen dorsal view **32** Frontal view **33** Endites and labium **34–35** Epigyna **36** Vulva. (arrows in Figs **29** & **31** indicate white spots being distinctive diagnostic characters). Abbreviations: cd: copulatory duct, co: copulatory opening, fd: fertilisation duct, s: spermatheca. Scale bars: 1 mm (**28–33**); 0.08 mm (**35**); 0.10 mm (**36**).

#### Etymology.

For Dr Robert Raven (Queensland Museum, Brisbane), distinguished Australian arachnologist and collector of the material studied.

#### Diagnosis.

Abdomen with white dorsal and ventral spots (Figs 29–31). Copulatory openings oriented anteriorly (Fig. 35), copulatory ducts undulating (Fig. 36).

#### Description.

Female holotype. Cephalothorax brown, with darker cephalic part, covered by sparse whitish scales. Foveal depression well marked (Fig. 28). Abdomen ovoid, grey brown with lighter pattern as in Fig. 31, covered with sparse brown hairs. Anterior spinnerets light brown, posterior ones whitish. Palps brown. Legs I brown, others lighter. Chelicerae brown, retromarginal tooth 5-cuspidate (Fig. 33). Labium and endites light brown, with lighter chewing margins. Sternum brown. Venter with white and greyish brown pattern (Fig. 30). Epigyne with copulatory openings well separated from each other and from spermathecae, the last in diagonal position, C-shaped (Figs 34–36). Dimensions. CL 1.30, CW 1.03, CH 0.60, EFL 0.60, AEW 1.00, PEW 0.95, AL 1.50, AW 1.07, LI: 4.05, LII: 2.85, LIII: 2.70, LIV: 3.15.

Male unknown.

#### Distribution.

Known from type locality only (Fig. 44).

### Rogmocrypta rollardae sp. n.

Taxon classificationAnimaliaAraneaeSalticidae

http://zoobank.org/94431F06-A47C-4CBA-936B-DE925E98083D

Figures 37–43 , 44

#### Material.

1♀ holotype, New Caledonia: Mandjélia (20°24'S, 164°32'E), 650m elev., rainforest, litter, berlesate, 12 May 1984, Monteith G, Cook D, QM S35648; 2♀ paratypes, New Caledonia: 4 km N of Col d’Amieu (21°19'48"S, 165°30'E), rainforest, litter, 300 m elev., berlesate No 640, 8 May 1984, Monteith G, Cook D, QM; 1♀, New Caledonia: Mandjélia (20°24'S, 164°32'E), 700 m elev., rainforest, litter, berlesate nr 648, 12 May 1984, Monteith G, Cook D, QM S35651; 1♀ New Caledonia: Dent de St. Vincent (166°13'02"E, 21°52'12"S), arete S, 1150 m elev., forêt-magius haut humide, berlese, 6 August 1987, A&S Tillier, Bonnet, Letocart, MNHN.

**Figures 37–43.**
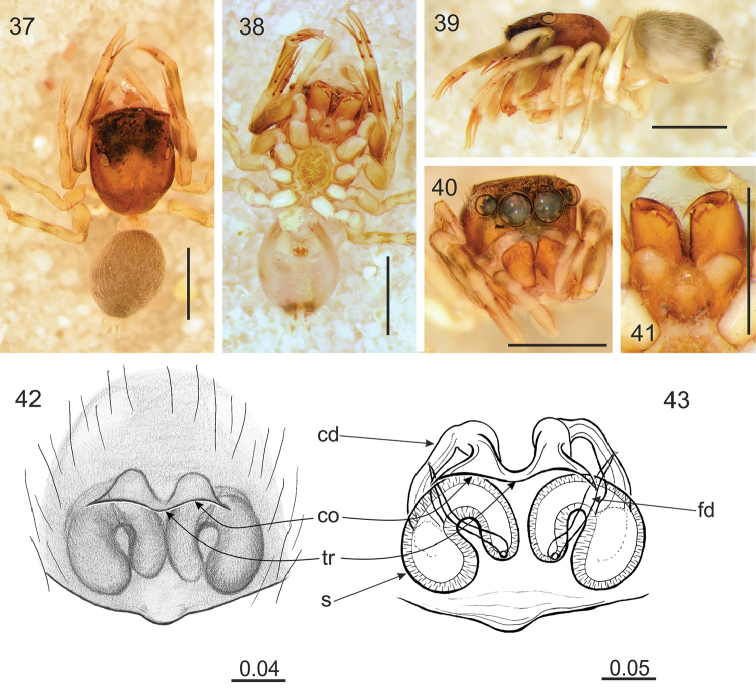
*Rogmocrypta rollardae* sp. n. (female holotype). **37** Dorsal view **38** Ventral view **39** Lateral view **40** Frontal view **41** Endites and labium **42** Epigyna **43** Vulva. Abbreviations: cd: copulatory duct, co: copulatory opening, fd: fertilisation duct, s: spermatheca, tr: transverse ridge. Scale bars: 1 mm (**37–41**); 0.04 mm (**42**); 0.05 mm (**43**).

#### Etymology.

For Dr. Christine Rollard (MNHN, Paris), distinguished French arachnologist.

#### Diagnosis.

Copulatory openings close to spermathecae, oriented posteriorly and joined, forming kind of transverse ridge (Figs 42–43).

#### Description.

Female holotype. Cephalothorax brown, covered with sparse white scales and brown hairs. Foveal depression well marked. Abdomen brownish, with lighter chevrons (Fig. 37). Anterior spinnerets light brown, posterior ones whitish. Palps and legs brownish with darker bands. Chelicerae brown, retromarginal tooth 6-cuspidate. Labium and endites light brown with lighter chewing margins. Sternum grey brown. Venter with white and grey brown pattern (Fig. 38). Epigyne with copulatory openings strongly sclerotized and close to each other (Figs 42–43). Copulatory ducts and spermathecae semicircular, the latter in diagonal position (Fig. 43). Dimensions. CL 1.82, CW 1.32, CH 0.83, EFL 0.78, AEW 1.22, PEW 1.09, AL 1.97, AW 1.45, LI: 5.24, LII: 3.64, LIII: 4.34, LIV: 4.92.

Male unknown.

#### Distribution.

**Figure 44.**
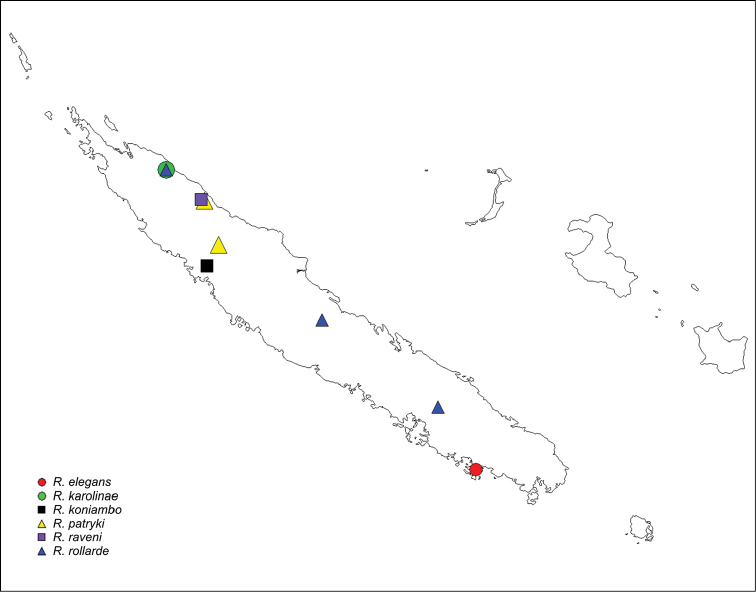
Distribution records of *Rogmocrypta*: *R. elegans* (red circle), *R. karolinae* (green circle), *R. koniambo* (black square), *R. patryki* (yellow triangle), *R. raveni* (purple square), *R. rollardae* (blue triangle).

Known from Mandjélia, Col d’Amieu and Dent de St. Vincent in New Caledonia (Fig. 44).

**Figure 45.**
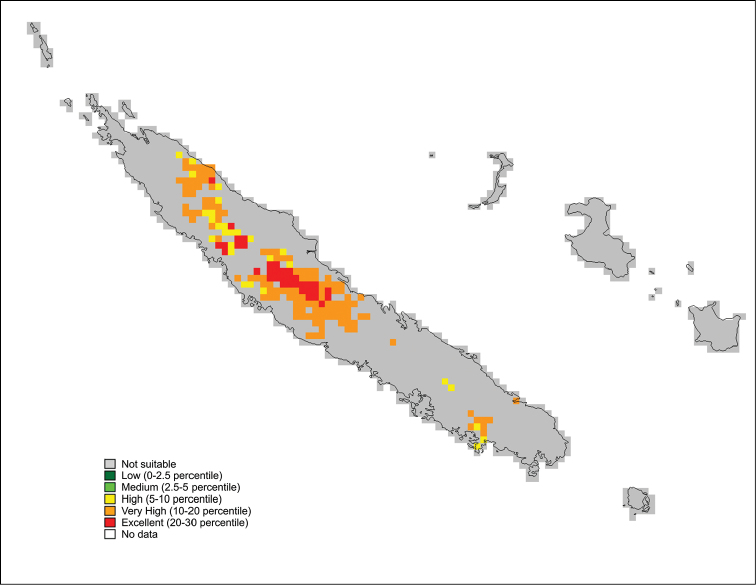
Predicted distribution of *Rogmocrypta* (14 records).

## Supplementary Material

XML Treatment for Rogmocrypta

XML Treatment for Rogmocrypta elegans

XML Treatment for Rogmocrypta karolinae

XML Treatment for Rogmocrypta koniambo

XML Treatment for Rogmocrypta patryki

XML Treatment for Rogmocrypta raveni

XML Treatment for Rogmocrypta rollardae
